# Dolutegravir in pregnant mice is associated with increased rates of fetal defects at therapeutic but not at supratherapeutic levels

**DOI:** 10.1016/j.ebiom.2020.103167

**Published:** 2020-12-18

**Authors:** Haneesha Mohan, Monica Guzman Lenis, Evelyn Y. Laurette, Oscar Tejada, Tanvi Sanghvi, Kit-Yi Leung, Lindsay S. Cahill, John G. Sled, Paul Delgado-Olguín, Nicholas D.E. Greene, Andrew J. Copp, Lena Serghides

**Affiliations:** a Toronto General Hospital Research Institute, Princess Margaret Cancer Research Tower (PMCRT), University Health Network, 101 College Street, 10th Floor, Room 359, Toronto, Ontario M5G 1L7, Canada; b Developmental Biology & Cancer Department, UCL Great Ormond Street Institute of Child Health, University College London, London, UK; c Mouse Imaging Centre, The Hospital for Sick Children, Toronto, Ontario, Canada; d Department of Chemistry, Memorial University of Newfoundland, St John's, Newfoundland and Labrador, Canada; e Department of Medical Biophysics, University of Toronto, Toronto, Ontario, Canada; f Translational Medicine, The Hospital for Sick Children, Toronto, Ontario M5G 0A4, Canada; g Department of Molecular Genetics, University of Toronto, Toronto, Ontario M5S 1A8, Canada; h Heart & Stroke Richard Lewar Centre of Excellence, Toronto, Ontario M5S 3H2, Canada; i Department of Immunology and Institute of Medical Sciences, University of Toronto, Toronto, Ontario, Canada; j Women's College Research Institute, Women's College Hospital, Toronto, Ontario, Canada

## Abstract

**Background:**

Dolutegravir (DTG) is a preferred regimen for all people with HIV including pregnant women, but its effects on the fetus are not fully understood. Periconceptional exposure to DTG has been associated with increased rates of neural tube defects (NTDs), although it is unknown whether this is a causal relationship. This has led to uncertainty around the use of DTG in women of reproductive potential.

**Methods:**

Pregnant C57BL/6J mice were randomly allocated to control (water), 1x-DTG (2.5 mg/kg-peak plasma concentration ~3000 ng/ml – therapeutic level), or 5x-DTG (12.5 mg/kg-peak plasma concentration ~12,000 ng/ml – supratherapeutic level), once daily from gestational day 0.5 until sacrifice. DTG was administered with 50 mg/kg tenofovir+33.3 mg/kg emtricitabine. Fetal phenotypes were determined, and maternal and fetal folate levels were quantified by mass-spectrometry.

**Findings:**

352 litters (91 control, 150 1x-DTG, 111 5x-DTG) yielding 2776 fetuses (747 control, 1174 1x-DTG, 855 5x-DTG) were assessed. Litter size and viability rates were similar between groups. Fetal and placenta weights were lower in the 1x-DTG vs. control. Placental weight was higher in the 5x-DTG vs. control. Five NTDs were observed, all in the 1x-DTG group. Fetal defects, including microphthalmia, severe edema, and vascular/bleeding defects were more frequent in the 1x-DTG group. In contrast, defect rates in the 5x-DTG were similar to control. Fetal folate levels were similar between control and 1x-DTG, but were significantly higher in the 5x-DTG group.

**Interpretation:**

Our findings support a causal relationship of DTG at therapeutic doses with increased risk for fetal defects, including NTDs at a rate that is similar that reported in the Tsepamo study for women exposed to DTG-based ART from conception. The non-monotonic dose-response relationship between DTG and fetal anomalies could explain the previous lack of fetal toxicity findings from pre-clinical DTG studies. The fetal folate levels suggest that DTG is unlikely to be an inhibitor of folate uptake.

**Funding:**

This project has been funded with Federal funds from the Eunice Kennedy Shriver National Institute of Child Health and Human Development, National Institutes of Health, Department of Health and Human Services, under Contract No. HHSN275201800001I.

**Research in context**

**Evidence before this study**

While dolutegravir (DTG) is now the recommended regimen for all people living with HIV due to its considerable advantages, the Tsepamo study neural tube defect (NTD) safety signal and the regulatory responses that followed the initial report of the signal, have led to uncertainty around the use of DTG in women of reproductive potential.

We searched PUBMED for “dolutegravir AND neural tube defects” up to May 2020 and found 30 publications. These included two reports from the Tsepamo birth surveillance study reporting the initial observation of a 0.94% relative risk for NTD in women receiving DTG-based antiretroviral therapy (ART) from conception vs. 0.12% with non-DTG-ART from conception, followed by the reduction of this risk to 0.3% vs. 0.1% respectively in 2019. The prevalence of NTD was higher in association with DTG vs. non-DTG ART at conception (0.2 percentage points difference (95% CI 0.01–0.59)). Major external structural defects were also higher in the DTG-ART from conception group (0.95% vs. 0.68% for non-DTG ART from conception), but this did not reach significance. The latest report from Tsepamo presented at the International AIDS Society 2020 meeting showed a risk of 0.19% for NTD with DTG vs. 0.11% for non-DTG ART exposure from conception. Several other reports outside of the Tsepamo surveillance study and Botswana reported no association between DTG and increased risk for NTDs, although these studies are underpowered to detect a signal. The WHO pharmacovigilance database (VigiBase) reported 17 cases of NTDs for DTG-containing regimens for an odds ratio of 6.4 (95% CI 3.7–10.9) compared with all other ARTs.

A second search for “dolutegravir AND (neural tube defects OR fetal anomalies) AND animal studies” found 3 results. One study examined dolutegravir in vitro and in a zebrafish model and reported that DTG is a noncompetitive folate receptor 1 antagonist, but not an inhibitor of dihydrofolate reductase at therapeutic concentration; early embryonic exposure to DTG was developmentally toxic in zebrafish, but could be mitigated by supplemental folate. In contrast, a second in vitro study reported that DTG is not a clinical inhibitor of folate transport pathways. A third pre-clinical developmental and reproductive toxicity study, testing DTG as a single drug at supratherapeutic doses, reported an absence of DTG-associated fetal defects. One NTD was seen in rabbits treated with 40 mg/kg DTG and one in rats treated with 1000 mg/kg DTG, although both were deemed to be not related to DTG.

**Added value of this study**

We performed a large prospective mouse DTG fetotoxicity study mimicking the clinical scenario as closely as possible. We evaluated gross fetal anomalies in mice randomly assigned to control (water), DTG-based ART (DTG in combination with tenofovir/emtricitabine) at doses yielding therapeutic plasma DTG levels (1x-DTG), or DTG-based ART at doses yielding supra-therapeutic DTG levels (5x-DTG) administered orally, once daily, throughout pregnancy. Considering that folic acid supplementation is common during pregnancy, we chose to feed mice a folate-sufficient diet to better simulate this situation. We evaluated fetuses (*n*=2776) for anomalies including NTDs on gestational day 15.5. We observed a 2-fold increased risk of fetal anomalies in mice exposed to therapeutic levels of DTG. These included 5 NTD, for a mean litter rate of 0.47% compared to 0% in the control group. Rates of eye defects, severe edema, and vascular defects were also 2 to 3-fold higher in mice exposed to therapeutic levels of DTG compared to controls. Surprisingly, the rates of fetal defects were similar between the 5x-DTG and control groups. We also measured total fetal folate levels by mass-spectrometry and found levels were significantly higher in the 5x-DTG group compared with the 1x-DTG and control groups, which did not differ from each other.

**Implications of all the available evidence**

The results of this study support a causal relationship of DTG at therapeutic doses with increased risk for fetal defects, including NTDs. Together with the Tsepamo findings, the data suggest that DTG from conception is associated with a small increase in the risk for NTDs. The non-monotonic dose-response relationship between DTG and fetal anomalies in our study could explain the previous lack of fetal toxicity findings from pre-clinical DTG studies, which used supratherapeutic dose levels. Our fetal folate level findings suggest that DTG is unlikely to be an inhibitor of folate uptake, in agreement with previous *in vitro* findings, although folate supplementation would likely be protective against NTDs in the context of DTG use in pregnancy.

Alt-text: Unlabelled box

## Introduction

Dolutegravir (DTG), an integrase strand transfer inhibitor, is an efficacious, well-tolerated drug with a high barrier to resistance [1]. In 2016, DTG became the World Health Organization (WHO) preferred first-line regimen for all people with HIV, although caution was noted about the lack of sufficient data for DTG use in pregnancy [2]. A significant increase in the risk for neural tube defects (NTDs) in infants born to women receiving DTG-based antiretroviral therapy (ART) from conception was reported in 2018 from the Tsepamo nationwide birth surveillance programme in Botswana [3]. With ongoing surveillance, there has been a period of decline since the original safety signal, with stabilization at a lower prevalence rate [4]. In July 2020, the prevalence for NTD with DTG-based ART at conception was 0.19% (95%CI 0.09–0.40), compared to 0.11% (95%CI 0.07–0.17) with non-DTG ART at conception and 0.07% (95%CI 0.06–0.09) in HIV-negative women [5]. In a study by the Botswana Ministry of Health, NTD prevalence was 0.65% with DTG-based ART at conception compared to 0% with non-DTG ART at conception and 0.09% in HIV-negative women [6]. Publications from other countries have not reported NTD with DTG exposures at conception, but have been limited by small sample sizes [[7], [8], [9], [10], [11], [12], [13]]. The largest database outside Botswana, the Antiretroviral Pregnancy Registry, has reported one NTD among 382 DTG preconception exposures, a prevalence of 0.26% [14]. The WHO pharmacovigilance database reported 17 cases of NTDs for DTG-containing regimens for an odds ratio (OR) of 6.4 (95%CI 3.7–10.9) compared with all other ARTs [15].

Drugs that are known human teratogens often show teratogenic defects in animal models, although not always [16,17]. Pre-clinical reproductive toxicology studies conducted on rats and rabbits found no DTG-related effects on fertility, embryonic, or fetal development [18], although these studies did not mimic the clinical scenario as they included higher than clinically relevant DTG doses (40–1000 mg/kg/day), tested DTG alone and not as part of an ART regimen, and exposed animals to DTG in specific windows that did not include the entire pregnancy [18]. Given the uncertainty surrounding the link between DTG and NTDs in humans, and the limitations of the pre-clinical DTG fetotoxicity studies, further animal studies modeling the clinical scenario are merited.

Here we present our findings from a large prospective mouse DTG fetotoxicity study where we evaluated gross fetal anomalies in pregnant mice randomly assigned to control, or DTG-based ART at doses yielding therapeutic, or supra-therapeutic DTG levels [19].

## Methods

*Ethics:* Animal experiments were approved by the University Health Network Animal Use Committee (#2575.25) and performed according to Canadian Council on Animal Care guidelines.

*Mice:* C57BL/6J mice bred in-house (original breeders from Jackson Laboratory RRID:IMSR JAX:000664), were maintained under a 12-h light/dark cycle, with *ad libitum* access to food and water. Mice were fed a standard folate-sufficient laboratory diet containing 7 mg/kg of folate (Teklad LM-485 Mouse/Rat Sterilizable Diet #7012). The relatively high levels of folate in the diet are meant to ensure adequate folate levels following diet sterilization (2–3 mg/kg). Animals were acclimated to their surroundings for 1 week prior to experiment initiation. Female mice (8–12 weeks of age) were trained on gavage with water prior to mating to reduce the potential stress during pregnancy, and were mated with males at a ratio of 2:1. Presence of vaginal plug was denoted as gestational day (GD) 0.5. Plugged females were randomly assigned to a treatment arm and housed in cages with 4 dams/cage. Pregnancy was confirmed by weight gain on GD9 (>1.5 g). All animal experiments were approved by the University Health Network Animal Use Committee (protocol #2575.25) and performed according to the policies and guidelines of the Canadian Council on Animal Care.

**Fig. 1:**
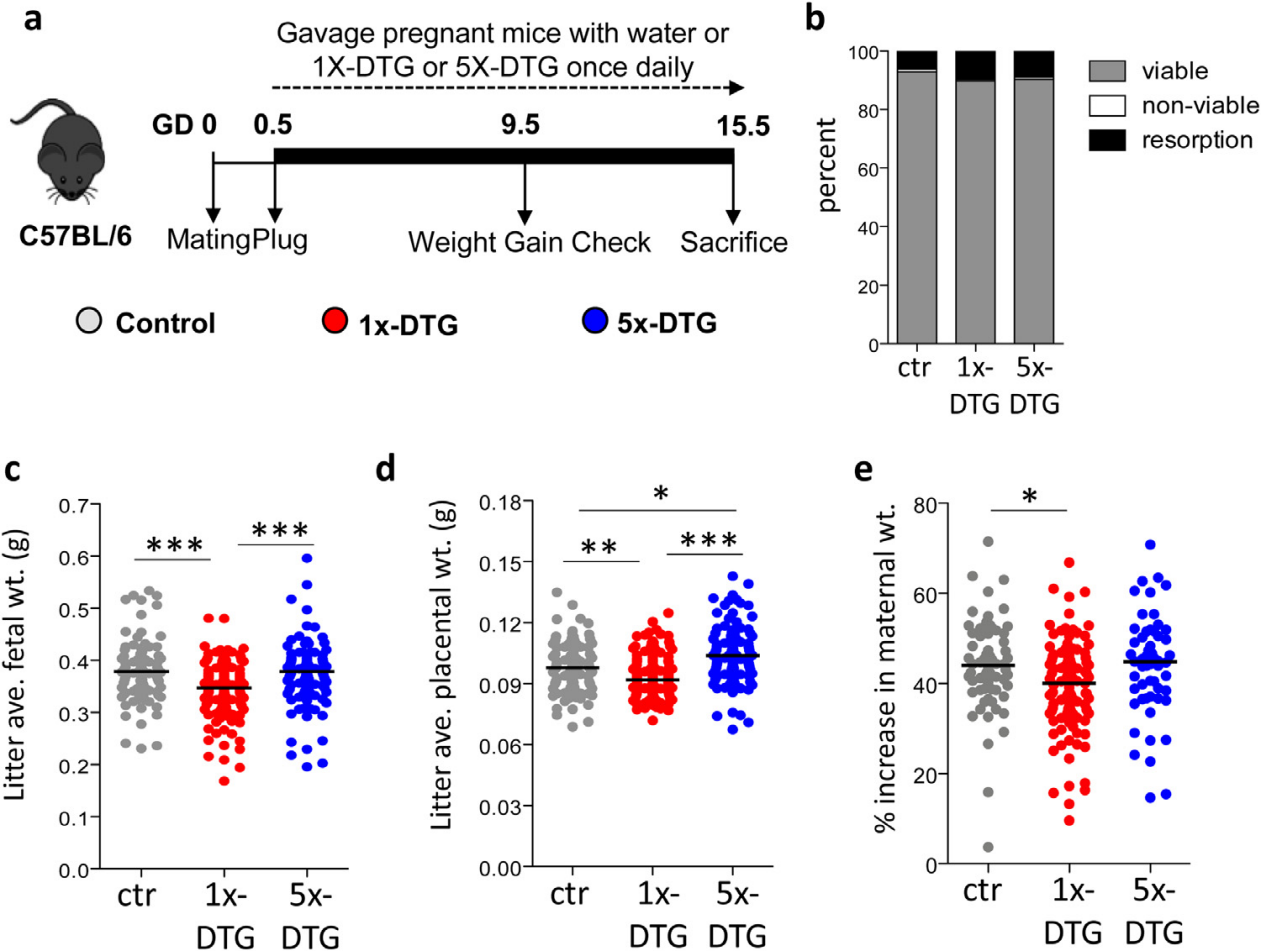
Comparison of pregnancy outcomes between treatment arms. Pregnant mice were treated with 1x-DTG (2.5 mg/kg DTG + TDF/FTC), 5x-DTG (12.5 mg/kg DTG + TDF/FTC), or water as a control starting on gestational day (GD) 0.5 until sacrifice on GD15.5. Schematic representation of the pregnancy mouse model is shown in **(a)**, viability rate in **(b)**, litter average fetal weight in **(c)**, litter average placental weight in **(d)**, and percent increase in maternal weight in **(e)**. Data are shown as dot plots with the line indicating the median. Statistical comparison by Kruskal–Wallis with Dunn's post-test vs control. * *p*<0.05, ** *p*<0.01, *** *p*<0.001. *N*=90 for control, *N*=143 for 1x-DTG, and *N*=100 for 5x-DTG.

*Treatment:* Dolutegravir (DTG) and tenofovir/emtricitabine (TDF/FTC) were purchased as prescription drugs. Pills were crushed, suspended in distilled water, and administered once daily by oral gavage (100 µL/mouse). Drug suspensions were prepared fresh each morning. Drug dosing was selected to yield maternal plasma levels equivalent to those reported in pregnant women, as determined in pilot studies [19]. The 1x-DTG group received 2.5 mg/kg DTG + 50 mg/kg TDF + 33.3 mg/kg FTC, yielding DTG peak plasma concentration of ~3000 ng/ml, Ctrough ~ 150 ng/mL. The 5x-DTG group received 12.5 mg/kg DTG + 50 mg/kg TDF + 33.3 mg/kg FTC, yielding DTG peak plasma concentration of ~12,000 ng/ml, Ctrough ~ 250 ng/mL. Control mice received 100 µL/mouse of distilled water once daily. On the day of plug detection (GD0.5), plugged mice were randomly allocated to control (water; *n*=91 litters, 747 fetuses), 1x-DTG (*n*=150 litters, 1174 fetuses) and 5x-DTG (*n*=111 litters, 855 fetuses), and were treated once daily until sacrifice (Fig. 1).

*Sample size calculation:* Sample size was decided based on a retrospective examination of banked fetuses from previous experiments completed in our laboratory that resulted in identification of two NTDs out of 158 fetuses (1.3%) exposed in utero to DTG-based ART, versus no NTDs in 187 control fetuses, and no NTDs in 64 fetuses exposed in utero to protease inhibitor-based ART. Based on these data we calculated a sample size of 750 fetuses per arm would give us a power of 0.8 to detect a significant difference between groups. Given the average litter size for a C57BL/6J mouse we estimated we would need 90–120 dams per group.

*Fetal collection and assessment:* Dams were euthanized at GD15.5 by CO_2_ inhalation and weight was recorded. A small number of dams were sacrificed on GD14.5 or 16.5 (7/150 in the 1x-DTG, 11/111 in the 5x-DTG, and 1/91 in the control). On day of sacrifice the uterus was surgically removed and the number and location of fetuses and resorptions (residues from early fetal demise, equivalent to spontaneous abortion) was recorded. The uterus was cut to separate each conceptus, and each fetus was carefully released into a dish containing PBS by tearing the amniotic sac. The placenta was also collected. Fetal viability was assessed by pedal reflex. Fetal and placental weights were recorded using a digital scale. Fetuses were examined under a stereo microscope, digital images were taken, and fetuses were fixed in 10% formalin. Images were scored for fetal macroscopic malformations by two independent investigators blinded to the treatment allocation (LS and AC). Disagreements between reviewers were resolved by discussion prior to unblinding. Select fetuses with anomalies and randomly selected controls were paraffin embedded, sectioned, and stained with hematoxylin and eosin (H&E). Fetal sections were examined by a fetal pathologist blinded to treatment allocation. Some fetuses underwent magnetic resonance imaging [20].

*Magnetic Resonance Imaging:* After dissection, fetuses were soaked in Gadolinium contrast agent (2 mM ProHance (Bracco Diagnostics Inc., # 0270-1111)). Anatomical scans of the fetuses were acquired using a 7.0 T magnet (Varian Inc.) and a 3D T2-weighted fast-spin echo sequence using a cylindrical *k*-space acquisition (TR=350 ms, TE=12 ms, echo train length=6, four averages, field-of-view = 20 × 20 × 20 mm, matrix size = 504 × 504 × 630, isotropic resolution=40 μm) [20]. The liver was manually segmented in 3D using Display (MINC toolkit, McConnell Brain Imaging Centre).

*Folate measurements:* Folate analysis of whole embryos and maternal liver collected on GD11.5, was performed by UPLC-MS/MS as described previously [21,22]. Briefly, buffer containing 20 mM ammonia acetate, 0.1% ascorbic acid, 0.1% citric acid and 100 mM DTT at pH7 was added to tissues and sonicated for 10 s using a hand-held sonicator at 40% amplitude on ice. Protein was removed by precipitation with addition of 2 volumes of acetonitrile, mixing for two minutes and centrifugation for 15 min at 12,000 × g at 4 °C. Supernatants were transferred to fresh tubes, lyophilised and stored at −80 °C prior to analysis.

Lyophilized embryo and maternal liver samples were resuspended in 30 µl and 60 µl water (milli-Q) and centrifuged for 5 min at 12,000 × g at 4 °C. Supernatants were transferred to glass sample vials for UPLC-MS/MS analysis. Metabolites were resolved by reversed-phase chromatography using Acquity UPLC BEH C18 column (50 mm × 2.1 mm; 1.7 µm bead size, Waters Corporation, UK). Solvents for UPLC were: Buffer A, 5% methanol, 95% Milli-Q water and 5 mM dimethylhexylamine at pH 8.0; Buffer B, 100% methanol. The column was equilibrated with 95% Buffer A: 5% Buffer B. The sample injection volume was 20 μl for embryo and 15 µl for liver. The UPLC protocol consisted of 95% Buffer A: 5% Buffer B for 1 min, followed by a gradient of 5–60% Buffer B over 9 min and then 100% Buffer B for 6 min before re-equilibration for 4 min. The metabolites were eluted at a flow rate of 200 nl/min. The UPLC was coupled to a XEVO-TQS mass spectrometer (Waters Corporation, UK) operating in negative-ion mode using the following settings: capillary 2.5 kV, source temperature 150 °C, desolvation temperature 600 °C, cone gas flow rate 150 L/h and desolvation gas flow rate 1200 L/h. Folates were measured by multiple reaction monitoring (MRM) with optimized cone voltage and collision energy for precursor and product ions (as described in [23]). Analysis of the peak areas were carried out using MassLynx software, Waters Corporation.

*Statistical analysis:* Fetal and placental weights are presented as litter averages for litters collected at GD15.5. Defects are reported at the fetal level as frequencies (number of fetuses showing the defect), at the litter level as number of litters that include at least one fetus showing the defect (% of litters affected), and as the mean litter rate of fetuses showing the defect (calculated by averaging the percent of fetuses in each litter with the defect) [24]. Kruskal–Wallis test with Dunn's post-test was used to compare mean litter rates. ORs with 95% confidence intervals (CI) were calculated using mixed-effects logistic regression with treatment as a fixed effect and litter as a random effect. Analyses were performed using GraphPad Prism (Version 5.0, La Jolla, CA) and (STATA Version 13).

*Role of funding source:* The study design was approved by the Funder. The funders played no role in data collection, analysis, or interpretation of the data, or drafting of the manuscript. The corresponding author had full access to all the data in the study and had final responsibility for the decision to submit for publication.

## Results

### Lower fetal and placenta weights in 1x-DTG, but not in 5x-DTG group

Pregnant dams were randomly allocated to the 1x-DTG (*n*=150), 5x-DTG (*n*=111) or control groups (*n*=91). The 1x-DTG group received 2.5 mg/kg DTG suspended in water, which yielded peak plasma levels of ~3000 ng/ml. Given that albumin levels in female C57BL/6J are similar to those seen in pregnant women (~3 g/dL) [25], this dose should approximate human therapeutic levels of DTG, although we were only able to measure total and not free DTG plasma levels. The 5x-DTG group received 12.5 mg/kg DTG, which yielded peak plasma levels of ~12,000 ng/ml. Both DTG regimens were given in combination with 50/33 mg/kg TDF/FTC, a commonly used dual nucleoside reverse transcriptase inhibitor (NRTI) backbone. The control group received water. Treatments were administered daily from GD0.5 to GD15.5 when pregnancy and fetal outcomes were assessed (Fig. 1a). Drug administration was not associated with any overt signs of maternal toxicity and distress. Fetal viability, fetal resorption (spontaneous abortion equivalent), and fetal non-viability (stillbirth equivalent) rates (Fig. 1b), as well as litter size (median [intra-quartile range]: 8 [7–9] for all groups) were similar between groups. The average fetal weight per litter was significantly lower in the 1x-DTG group compared to control and 5x-DTG groups **(**Fig. 1c). Average placental weight per litter was lower in the 1x-DTG group compared to control, whereas placental weight was significantly higher in the 5x-DTG compared to control and 1x-DTG groups (Fig. 1d). Maternal weight gain during pregnancy was significantly lower in the 1x-DTG group compared to control, but did not differ between control and 5x-DTG (Fig. 1e).

### Neural-tube defects occur only in the 1x-DTG group

**Fig. 2:**
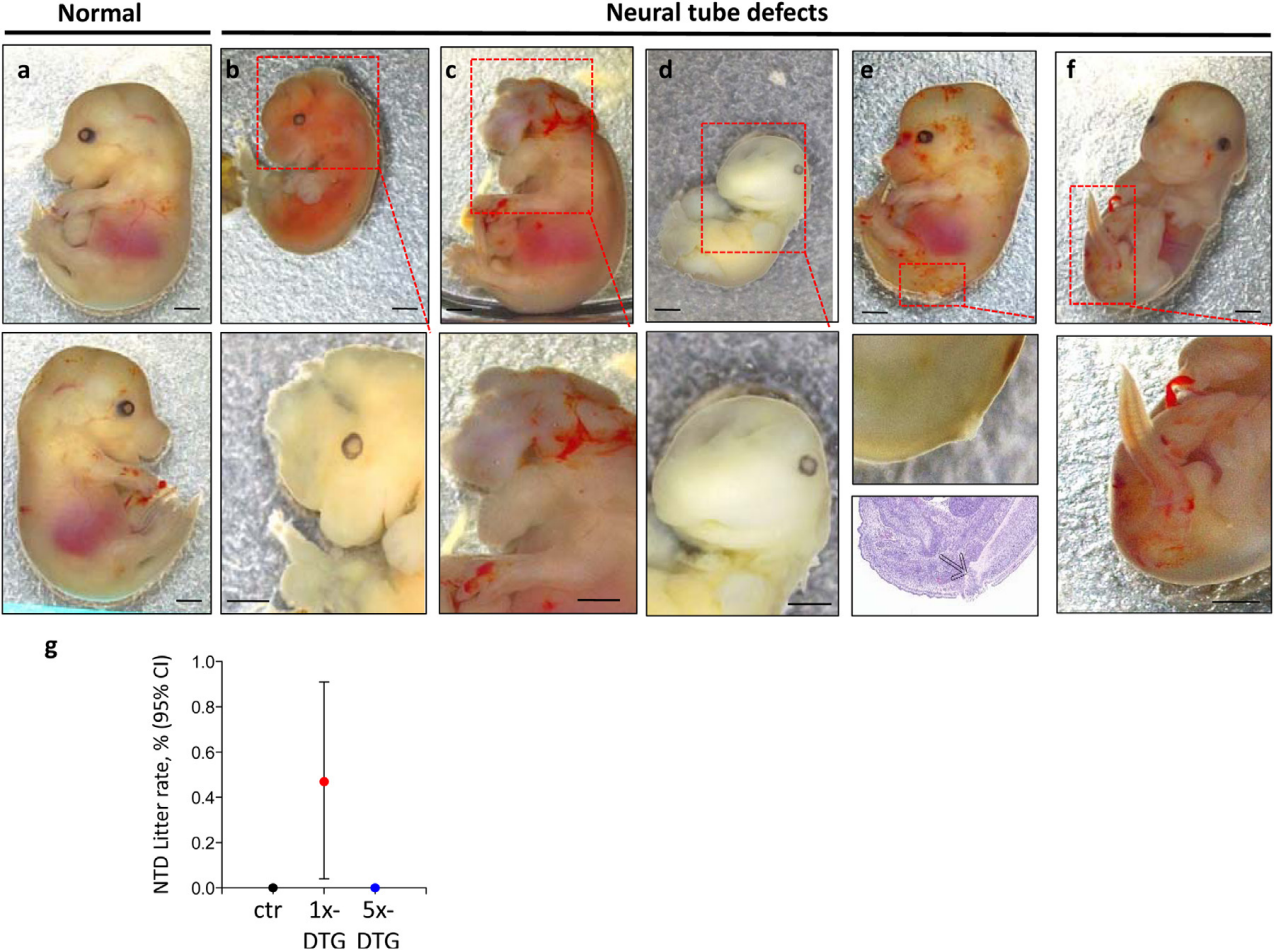
Neural tube defects in fetuses in the 1x-DTG treatment arm. Images of a representative control **(a)**, and the five fetuses with neural tube defects **(b–f)**. Scale bars = 1 mm. **(b)** Non-viable fetus exhibiting exencephaly, kinky tail, hemorrhage, and growth retardation. **(c)** Non-viable, edematous fetus exhibiting exencephaly, holoprosencephaly, mandibular aplasia, maxillary prognathism, and bilateral anophthalmia. **(d)** Non-viable fetus with open forebrain, and growth retardation. **(e–f)** Viable fetuses exhibiting spina bifida. The region of defect is highlighted by a dashed red box, with a higher magnification of the affected region in the lower images. An H&E section shows evidence of spina bifida in **(e)** bottom panel. **(g)** Mean litter rate with 95% confidence interval for neural tube defects in the control (ctr, *n*=91 litters), 1x-DTG (2.5 mg/kg DTG + TDF/FTC, *n*=150 litters) and 5x-DTG (12.5 mg/kg DTG + TDF/FTC, *n*=111 litters). Neural tube defects mean litter rate is significantly higher in the 1x-DTG group vs. control (*p*<0.05). Statistical comparison by Kruskal–Wallis with Dunn's post-test.

Five fetuses from the 1x-DTG group exhibited NTDs (Fig. 2), giving an incidence rate of 0.43% (5/1174), while no NTDs were detected in the control (0/747) or the 5x-DTG groups (0/849). All 5 fetuses came from different litters. The mean litter rate for NTDs in the 1x-DTG group (calculated by averaging the percent of fetuses per litter with the defect [24]) was 0.47%, compared to 0% in the control and 5x-DTG groups (Fig. 2g). Three of the fetuses were non-viable. The first fetus exhibited exencephaly and a kinked tail (indicator of spinal dysraphism [26]), was hemorrhagic, and growth retarded (Fig. 2b). The second fetus exhibited exencephaly (protruding brain tissue), holoprosencephaly (failure of the brain to divide into two hemispheres), and facial anomalies including mandibular aplasia (shortening of lower jaw), maxillary prognathism (abnormal protrusion of upper jaw), bilateral anophthalmia (lack of both eyes), and was edematous (Fig. 2c). The third fetus was growth retarded and had an opening in the forebrain region. Follow-up examination demonstrated forebrain agenesis indicating a potential anencephaly (Fig. 2d). The last two fetuses exhibited open spina bifida (Fig. 2e and f). The OR for NTDs was 6.92 (95%CI 0.38–127) for the 1x-DTG compared to control, which did not reach statistical significance (*p*=0.16, by Fisher's exact test).

### Fetal defects are more frequent in the 1x-DTG group

To determine if maternal exposure to DTG led to a higher frequency of other fetal defects besides NTDs, we examined all fetuses for gross anomalies and calculated ORs compared to control for each fetal anomaly with more than 10 occurrences, adjusting for litter effects. The mean litter rate for the presence of any defect was 17.7% in the control, 30.2% in the 1x-DTG (*p*<0.001 vs. control, by Kruskal–Wallis test with Dunn's multiple comparison post-test), and 19.2% in the 5x-DTG groups (Table 1). The OR for any fetal defect was ~2-fold higher in the 1x-DTG compared to control (litter adjusted OR (aOR) 1x-DTG *vs*. control: 2.04 (95%CI 1.54–2.69)). The aOR for any fetal defect was similar between the 5x-DTG and control groups (Table 2).

**Table 1:** Congenital fetal anomalies in mice exposed to either 1x-DTG, 5x-DTG, or control.

	Control *N* (litter) = 91, *N* (fetus) = 747			1x-DTG *N* (litter) = 150, *N* (fetus) = 1174				5x-DTG *N* (litter) = 111, *N* (fetus) = 849		
	Fetuses affected *n* (%)	Litters affected *n* (%)	Mean litterRate % (95% CI)	Fetuses affected *n* (%)	Litters affected *n* (%)	Mean litter rate % (95% CI)	Fetuses affected *n* (%)		Litters affected *n* (%)	Mean litter rate % (95% CI)
**Any defect**	133 (17.8%)	65 (71.4%)	17.7% (14.2, 21.3)	349 (29.7%)	129 (86%)	**30.2%**^a^*** (26.7, 33.7)	154 (18.1%)		78 (70.3%)	19.2% (15.7, 22.7)
**Neural tube defects**										
Open neural tube defects	0 (0%)	0 (0%)	0% (0, 0)	5^b^ (0.43%)	5 (3.3%)	0.47%* (0.04, 0.91)	0 (0%)		0 (0%)	0% (0, 0)
Tail flexion, kinked tail, curled tail	12 (1.61%)	12 (13.2%)	1.84% (0.74, 2.93)	27 (2.3%)	22 (14.7%)	2.19% (1.24, 3.14)	19 (2.24%)		13 (11.7%)	13 (11.7%)
**Face, ear, and neck defects**										
Any face/ear/neck anomaly	3 (0.4%)	3 (3.3%)	0.44% (−0.067, 0.95)	13 (1.11%)	10 (6.7%)	1.09% (0.34, 1.84)	4 (0.47%)		4 (3.6%)	0.5% (0.001, 1.0)
Facial anomaly	2 (0.27%)	2 (2.2%)	0.32% (−0.13, 0.77)	8^c^ (0.68%)	8 (5.3%)	**0.67%^#^ (0.20, 1.41)**	0 (0%)		0 (0%)	0% (0, 0)
Oro-facial clefts	1 (0.13%)	1 (1.1%)	0.12% (−0.12, 0.36)	1 (0.09%)	1 (0.7%)	0.11% (−0.11, 0.33)	0 (0%)		0 (0%)	0% (0, 0)
Mandibular aplasia	0 (0%)	0 (0%)	0% (0, 0)	6^d^ (0.51%)	5 (3.3%)	0.61% (−0.014, 1.24)	4 (0.47%)		4 (3.6%)	0.50% (0.001, 1.0)
**Eye defects**										
Any eye defect	25 (3.35%)	19 (20.9%)	3.29% (1.50, 5.10)	72 (6.13%)	56 (37.3%)	5.90%* (4.48, 7.32)	39 (4.59%)		32 (28.8%)	4.40% (2.98, 5.82)
Anophthalmia	3 (0.40%)	2 (2.2%)	0.38% (−0.17, 0.94)	8 (0.68%)	5 (3.3%)	0.46% (0.048, 0.87)	4 (0.47%)		4 (3.6%)	0.71% (−0.048, 1.46)
Microphthalmia	9 (1.20%)	8 (8.8%)	1.24% (0.35, 2.12)	41 (3.49%)	36 (24%)	3.49%** (2.40, 4.58)	21 (2.47%)		20 (18%)	2.65% (1.50, 3.80)
Other eye defects	14 (1.87%)	11 (12.1%)	1.81% (0.58, 3.05)	32 (2.73%)	30 (20%)	2.70% (1.76, 3.64)	23 (2.71%)		19 (17.1%)	2.28% (1.28, 3.28)
**Abdominal wall defects**										
Gastroschisis, omphalocele	1 (0.13%)	1 (1.1%)	0.14% (−0.14, 0.41)	4 (0.34%)	4 (2.7%)	0.36% (−0.01, 0.72)	2 (0.24%)		2 (1.8%)	0.33% (−0.13, 0.79)
**Limb defects**										
Limb asymmetry, club foot	2 (0.27%)	2 (2.2%)	0.23% (−0.093, 0.56)	2 (0.18%)	2 (1.33%)	0.13% (−0.05, 0.30)	3 (0.35%)		3 (2.7%)	0.38% (−0.07, 0.83)
**Vascular/bleeding defects**										
Any vascular/bleeding defect	50 (6.69%)	31 (34.1%)	6.22% (4.09, 8.34)	169 (14.4%)	85 (56.7%)	15.3%*** (12.17, 18.43)	49 (5.77%)		32 (28.8%)	6.86% (4.03, 9.69)
Petechiae	38 (5.09%)	25 (27.5%)	4.62% (2.84, 6.41)	117 (9.97%)	69 (46%)	10.5%** (7.95, 13.03)	39 (4.59%)		30 (27%)	5.72% (3.12, 8.32)
Cranial/spinal bleeds	10 (1.34%)	9 (9.9%)	1.43% (0.46, 2.41)	50 (4.26%)	43 (28.7%)	4.68%*** (2.97, 6.39)	10 (1.18%)		7 (6.31%)	1.14% (0.25, 2.03)
Hemorrhagic	4 (0.54%)	4 (4.4%)	0.57% (0.002, 1.14)	12 (1.02%)	12 (8.0%)	**1.16%# (0.46, 1.86)**	1 (0.12%)		1 (0.9%)	0.11% (−0.11, 0.34)
**Other defects**										
Body asymmetry	16 (2.14%)	8 (8.8%)	1.85% (0.36, 3.34)	24 (2.04%)	19 (12.7%)	1.98% (1.04, 2.91)	18 (2.12%)		15 (13.5%)	1.8% (0.88, 2.72)
Severe edema	11 (1.47%)	8 (8.8%)	1.46% (0.42, 2.51)	45 (3.83%)	36 (24%)	3.78%** (2.53, 5.03)	10 (1.18%)		9 (8.1%)	1.03% (0.33, 1.74)

a For percent litter average: statistical comparisons between treatment groups by Kruskal–Wallis test with Dunn's multiple comparison post-test. * *p*<0.05, ** *p*<0.01, *** *p*<0.001 vs. control. ^#^*p*<0.05 vs. 5x-DTG.

b The five open neural tube defects were two exencephalies, one anencephaly, and two spina bifidas.

c Two fetuses had both a facial anomaly and mandibular aplasia. These are counted only once in the “any anomaly” category.

**Table 2:** Odds ratios for fetal anomalies in the DTG groups versus control.

	1x-DTG vs. control		5x-DTG vs. control	
	aOR^a^ (95%CI)	*p*-value	aOR^a^ (95%CI)	*p-*value
**Any defect**	2.04 (1.54, 2.69)	**<0.001**	1.04 (0.76, 1.42)	0.81
**Tail defects**	1.41 (0.64, 3.1)	0.40	1.34 (0.57, 3.14)	0.50
**Any face/ear/neck defect**	2.79 (0.73, 10.7)	0.13	1.18 (0.24, 5.73)	0.84
**Any eye defects**	1.97 (1.18, 3.31)	**0.01**	1.45 (0.82, 2.55)	0.20
**Anophthalmia**	1.83 (0.43, 7.86)	0.42	1.27 (0.25, 6.48)	0.77
**Microphthalmia**	2.97 (1.44, 6.14)	**0.003**	2.08 (0.95, 4.57)	0.068
**Other eye defects**	1.52 (0.76, 3.05)	0.24	1.52 (0.73, 3.18)	0.27
**Any vascular/bleed defect**	2.66 (1.65, 4.28)	**<0.001**	0.87 (0.50, 1.51)	0.62
**Petechiae**	2.30 (1.37, 3.87)	**0.002**	0.95 (0.53, 1.73)	0.87
**Cranial/spinal bleeds**	3.32 (1.63, 6.74)	**0.001**	0.88 (0.36, 2.16)	0.78
**Hemorrhagic**	1.92 (0.62, 5.97)	0.26	0.22 (0.02, 1.96)	0.18
**Body asymmetry**	1.04 (0.44, 2.47)	0.92	1.08 (0.43, 2.71)	0.86
**Severe edema**	2.76 (1.33, 5.73)	**0.007**	0.79 (0.31, 1.98)	0.62

a Adjusted odds ratios calculated using mixed effects logistic regression using treatment as a fixed effect and litter as a random effect.

Facial anomalies, oro-facial clefts, body asymmetry, abdominal-wall defects, limb defects, and abnormal tail phenotypes, including kinked tail, curled tail, and tail flexion (Fig. S1), were seen at similar rates across treatment groups (Table 1). There was a trend towards higher rates of mandibular aplasia in both the 1x-DTG (0.61%) and 5x-DTG (0.50%) compared to control (0%) (Table 1).

**Fig. 3:**
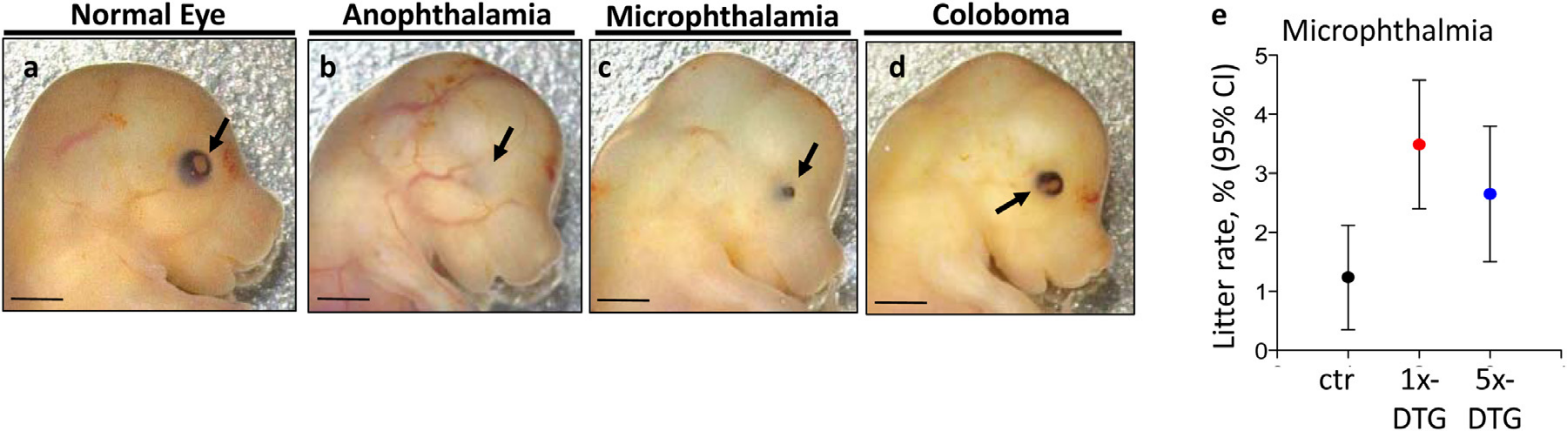
Eye defects are more common in fetuses in the 1x-DTG treatment arm. Gross morphology of representative fetuses showing normal eyes **(a)**, anophthalmia **(b)**, microphthalmia **(c),** and coloboma **(d)**. Arrows point to the eye. Scale bars = 1 mm. **(e)** Mean litter rate with 95% confidence interval for microphthalmia in the control (ctr), 1x-DTG (2.5 mg/kg DTG + TDF/FTC) and 5x-DTG (12.5 mg/kg DTG + TDF/FTC). Mean litter rate for microphthalmia is significantly higher in the 1x-DTG group vs. control (*p*<0.01). Statistical comparison by Kruskal–Wallis with Dunn's post-test. *N*=91 litters for control, *N*=150 litters for 1x-DTG, *N=*111 litters for 5x-DTG.

Eye defects included anophthalmia (complete absence of the eye), microphthalmia (small eye), and coloboma (malformed lens and incomplete formation of retinal pigment epithelium ring) (Fig. 3). Compared to control, fetuses in the 1x-DTG group were significantly more likely to display an eye defect (mean litter rate: 5.90% in 1x-DTG vs. 3.29% in control; aOR 1x-DTG vs. control: 1.97 (95%CI 1.18–3.31)) (Table 1). Among the different categories of eye defects, the rate of microphthalmia was significantly higher in the 1x-DTG group compared to control (Fig. 3e).

### Severe edema and vascular defects are more common in the 1x-DTG group

**Fig. 4:**
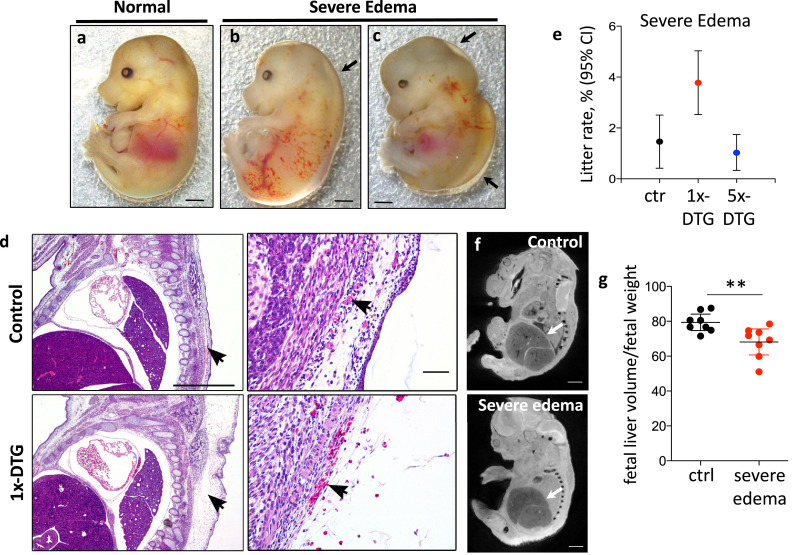
Severe edema is more common in fetuses in the 1x-DTG treatment arm and coincides with vascular leak and lower fetal liver volumes. Gross morphology of representative fetuses, normal/control **(a)**, and severe edema **(b–c)**. Scale bars = 1 mm. Black arrows highlight regions of edema **(b, c). (d)** H&E stained sections from a control fetus (top) and a 1x-DTG fetus (bottom). Arrows in the left panels point to the area of edema in a 1x-DTG fetus (bottom-left panel) and equivalent area in the control (upper-left panel). Arrows in close ups in the right panels point to areas of blood pooling in the 1x-DTG (lower-right panel) and equivalent area in the control (upper-right panel). Vascular leakage is evidenced by presence of erythrocytes within the edematous area in a 1x-DTG fetus (bottom-right panel). Scale bars = 1 mm and 100 µm in close ups. **(e)** Mean litter rate with 95% confidence interval for severe edema in the control (ctr), 1x-DTG (2.5 mg/kg DTG + TDF/FTC) and 5x-DTG (12.5 mg/kg DTG + TDF/FTC). Mean litter rate for severe edema is significantly higher in the 1x-DTG group vs. control and 5x-DTG (*p*<0.01). Statistical comparison by Kruskal–Wallis with Dunn's post-test. *N*=91 litters for control, *N*=150 litters for 1x-DTG, *N*=111 litters for 5x-DTG. **(f)** Mid-sagittal sections of fetal images acquired by magnetic resonance imaging of a control fetus (top panel) and a 1x-DTG fetus with severe edema (bottom panel). White arrows point to the liver. Scale bars = 1 mm. **(g)** Fetal liver volume (normalized to fetal weight) is lower in 1x-DTG fetuses with severe edema. Data shown as dot plots with mean and 95% confidence interval. Statistical comparisons by Student's *t*-test. *N*=8 fetuses/group from 8 different litters. ** *p*<0.01.

Fetuses in the 1x-DTG group were significantly more likely to display severe edema (Fig. 4a–e) compared to control (Table 1), with an aOR of 2.76 (95%CI 1.33–5.73). The mean litter rate for severe edema was 3.78% in the 1x-DTG, compared to 1.46% in the control and 1.03% in the 5x-DTG group (Fig. 4e). Magnetic resonance imaging analysis of a subset of fetuses revealed an association between severe edema and liver atrophy, with edematous fetuses having lower liver volumes compared to controls (Fig. 4f–g).

**Fig. 5:**
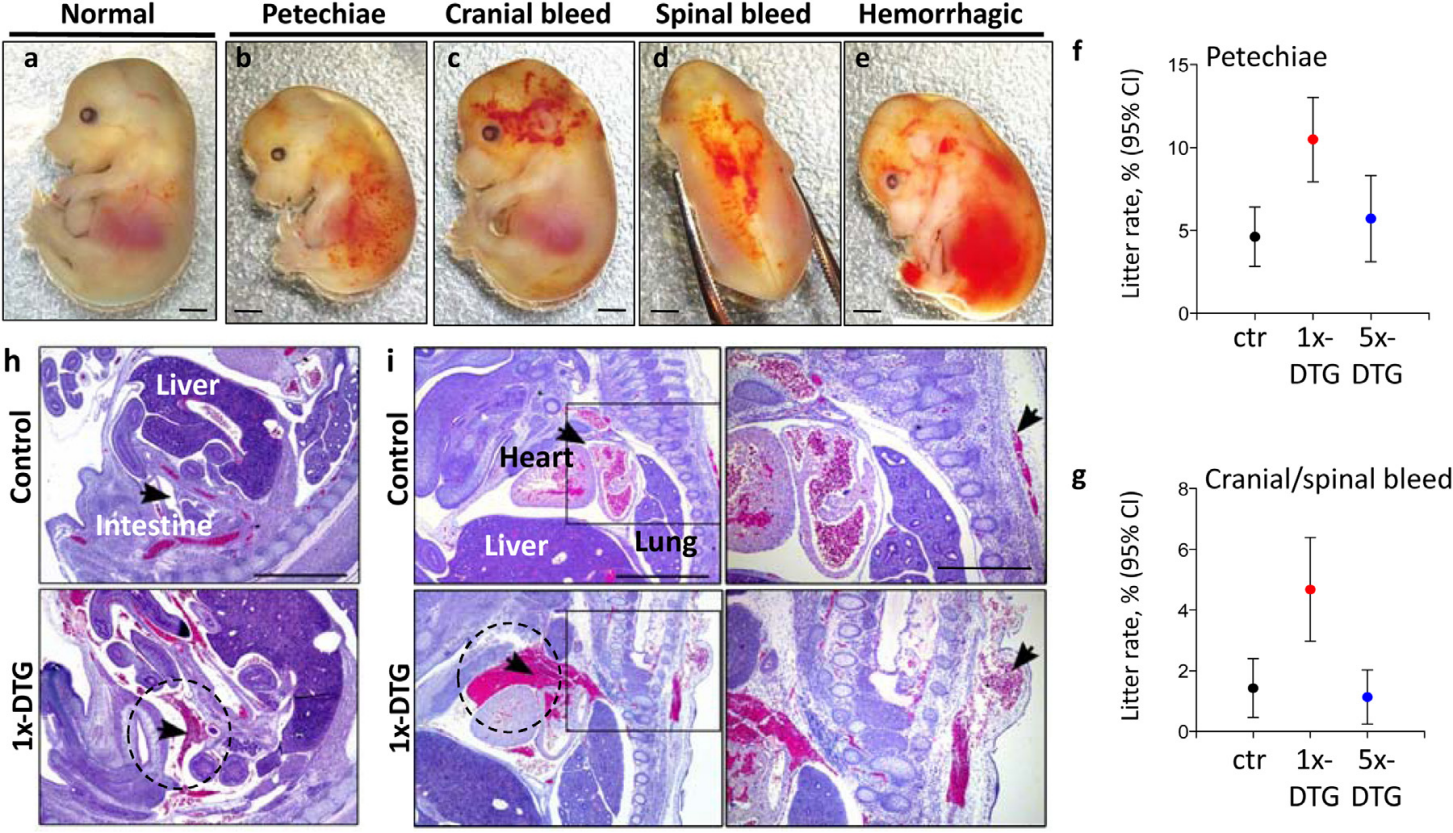
Vascular defects are more common in fetuses in the 1x-DTG treatment arm. Gross morphology of representative fetuses showing petechiae **(b)**, cranial bleed **(c)**, spinal bleed **(d)**, and hemorrhagic bleed **(e)**, compared with a normal fetus **(a)**. Scale bars = 1 mm. Mean litter rate with 95% confidence interval for petechiae in **(f)** and cranial or spinal bleeds in **(g)** in the control (ctr), 1x-DTG (2.5 mg/kg DTG + TDF/FTC) and 5x-DTG (12.5 mg/kg DTG + TDF/FTC). Both types of vascular disorder are significantly more prevalent in the 1X-DTG group vs. control (*p*<0.01 for petechial, *p*<0.001 for cranial/spinal bleed). Statistical comparison by Kruskal–Wallis with Dunn's post-test. *N*=91 litters for control, *N*=150 litters for 1x-DTG, *N*=111 litters for 5x-DTG. **(h–i)** H&E sections from control and 1x-DTG fetuses. **(h)** Intra-peritoneal hemorrhage in a 1x-DTG fetus, which also had a cranial bleed (as seen in C). **(i)** Pericardial hemorrhage in a 1x-DTG fetus which also had a spinal bleed (as seen in D). Close ups are shown in the right panels. Scale bar = 1 mm and 0.5 mm in close ups.

We also observed a higher mean litter rate of vascular defects in the 1x-DTG group (15.3%) compared to control (6.22%) and 5x-DTG (6.86%) (Table 1). These defects included the presence of petechiae-like superficial bleeding covering large areas of the body (Fig. 5b), or more localized to the head (Fig. 5c) and/or the back (Fig. 5d), and a more hemorrhage-like bleeding pattern (Fig. 5e). These defects were significantly more common in the 1x-DTG compared to control and 5x-DTG groups (Table 1, Fig. 5h and i). Fetuses with petechiae-like superficial bleeds were 2.3-fold (95%CI 1.37–3.87) more frequent in the 1x-DTG vs. control group (Fig. 5h), and fetuses with bleeds in the head or back were 3.3-fold (95%CI 1.63–6.74) more frequent in the 1x-DTG vs. control group (Tables 1 and 2). The frequency of vascular defects was similar between control and 5x-DTG.

To determine if 1x-DTG fetuses also had internal hemorrhages, we performed histological analyses. Fetuses with edema frequently displayed signs of vascular defects, with erythrocytes extravasating into edematous areas (Fig. 4d), suggesting a possible link between severe edema and vascular instability. Moreover, fetuses with superficial bleeds on the head or back had internal hemorrhage in the abdominal cavity (Fig. 5h) or in the pericardium (Fig. 5i).

### Higher fetal folate levels in the 5x-DTG group

**Fig. 6:**
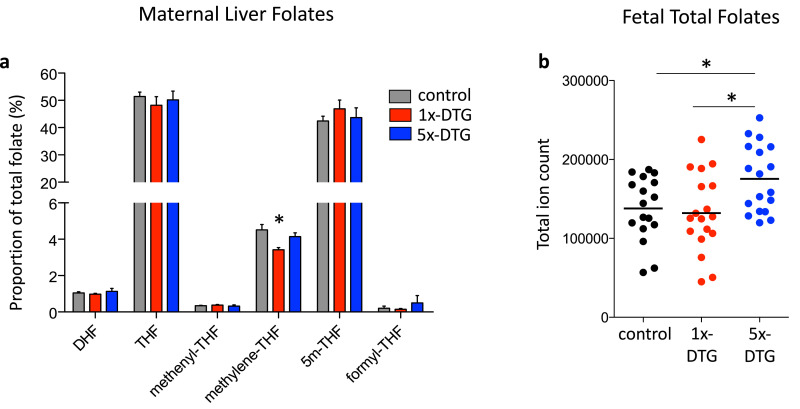
Maternal liver folate profiles and fetal total folate levels differ between treatment arms. Folate profile in maternal liver **(a)** and total folate levels in whole fetuses **(b)** collected on gestational day 11.5 and assessed by mass spectrometry. Statistical comparisons by one-way ANOVA with Bonferroni's multiple comparisons post-test. Maternal folate profiles differ only in a reduced methylene-THF proportion in the 1x-DTG group vs. control. Fetal total folates are significantly higher in the 5x-DTG group vs. control (* *p*<0.05 vs. control and 1x-DTG). For maternal folates *N*=6/group. For fetal folates *N*=18 fetuses/group from 6 different litters. Data are shown as means with SEM in (a) and as dots plots with the line indicating the mean in (b). DHF, dihydropholate; THF, tetrahydrofolate; 5m-THF, 5 methyltetrahydrofolate.

Folate status is an established modifier of risk for fetal anomalies, and NTDs in particular [[27], [28], [29]]. *In vitro* studies have identified a potential interaction between DTG and folate-receptor 1 (FOLR1) [30], with DTG acting as a partial antagonist of FOLR1 *in vitro* [31]. We compared maternal liver folate profiles and total fetal folate levels by mass-spectrometry methodology that allows analysis of unmetabolized folic acid and the six major folates, including their polyglutamated forms. Tissue was collected from control, 1x-DTG, and 5x-DTG treated mice at GD11.5, a time point which coincides with neural tube closure and allows for analysis of the entire fetus. Differences in maternal liver folate profiles were observed in the 1x-DTG group compared to control, with a significant reduction in methylene tetrahydrofolate as a proportion of total folate. In contrast, folate profiles were similar in control and 5x-DTG group (Fig. 6a). Fetal total folate levels were similar between control and 1x-DTG, whereas fetal total folate levels were significantly higher in the 5x-DTG compared to both 1x-DTG and control (Fig. 6b). This finding may indicate induction of a protective mechanism (i.e. enhanced folate status) for fetuses exposed to 5x-DTG. Fetal folate profiles were similar between groups with over 96% of fetal folates being 5-methyl-tetrahydrofolate (5m-THF) (Fig. S2). Unmetabolized folic acid was not detected in either maternal liver or fetal samples.

## Discussion

Here we report findings from a large DTG fetotoxicity study in mice. Compared to controls, we observe a 2-fold increased risk of fetal anomalies, as well as lower fetal and placenta weights, in mice exposed to a dose of DTG that yields therapeutic plasma levels, administered with a dual NRTI backbone consisting of TDF/FTC (1x-DTG). In contrast, a 5× higher dose of DTG resulted in a rate of fetal anomalies similar to that seen in the control group, an unexpected lack of a dose response, as well as higher placenta weights and no fetal growth restriction.

We observed 5 NTDs in our study, all occurring in the 1x-DTG group, for a mean litter rate of 0.47%, similar to the rate of 0.3% reported for women on DTG-based ART from conception in the Botswana-based surveillance study in 2019 [4] and the 0.19% rate reported in 2020 [5]. The types of NTDs we observed were diverse in nature, and spanned a similar range as observed in humans [4]. C57BL/6 is an inbred mouse strain that is resistant to NTDs, so the detection of even a small number of NTDs is notable, especially given that all our mice were fed a folate-sufficient diet. Previous studies reported one case of cranioschisis in rabbits administered 40 mg/kg DTG and one meningocele in rats administered 1000 mg/kg DTG [18]. Both were deemed unrelated to the drug. Differences between our study and these preclinical studies include our use of a lower dose of DTG, which was administered with an NRTI backbone, and our treatment of pregnant mice from GD0.5 while the preclinical studies began treatment post-implantation at GD6. The early start in treatment in our study mimics the clinical scenario better, as the prevalence of NTDs was higher in women with DTG exposure from conception, compared to those who initiated DTG later in pregnancy [3,4].

We observed higher rates of eye defects in the 1x-DTG group. Although, eye defects are commonly observed anomalies in C57BL/6 mice [32], the rates of eye defects were significantly higher in 1x-DTG group suggesting a drug-associated effect. Anophthalmia and microphthalmia often coincide with brain abnormalities, and have been associated with mutations in several genes that are also expressed in neural stem cells and play a role in neural tube development [[33], [34], [35]]. Our data may suggest a possible impact of DTG on neural stem cells, affecting eye and potentially neural tube and brain development. Of interest, an investigation of neurological outcomes in children born to women with HIV reported an increase in risk of having a neurological diagnosis, which included ophthalmological disorders, with *in utero* exposure to DTG particularly in the first trimester [36].

The most frequent defects observed in our study were severe edema, resembling fetal hydrops, and bleeding defects, both of which were more common in the 1x-DTG group compared to control, but were similar between the 5x-DTG group and control. Several mechanisms have been proposed to contribute to fetal hydrops including decreased plasma osmotic pressure, increased capillary permeability, and obstructed lymphatic flow, which could lead to abnormal water transport between the capillary plasma and extravascular tissues [37]. In histological examinations of fetuses with severe edema we observed evidence of extravasated erythrocytes suggesting that increased vascular permeability could have contributed to the severe edema. Liver dysfunction can also lead to severe edema and has been reported in cases of non-immune fetal hydrops [37]. We observed an association between severe edema and lower liver volumes, which could be indicative of poor liver development or function.

Defective vascular integrity may also contribute to the bleeding defects we observed especially in the 1x-DTG group, and may suggest that the edema and bleeding defects could be related. Edema (not always severe) coincided with bleeding defects. Histological examination of a subset of fetuses with bleeding defects revealed evidence of internal hemorrhage, suggesting that the bleeding defects maybe more pronounced than was evident through external examination. Thus, our data suggest that DTG (at least at the 1× dose) could have a negative effect on vascular integrity during fetal development. Future studies investigating the interaction between DTG and embryonic vascular development are merited.

Disruption of folate uptake or metabolism is a common pathway through which several drugs associated with congenital defects exert their teratogenic effects [38]. Folate sensitive birth defects include NTDs, orofacial clefts, and limb defects. A recent study of 406 women living with HIV initiating either a DTG or an efavirenz based regimen found no significant declines in serum folate concentrations associated with DTG treatment. However, a slight decrease was reported at 12 weeks of treatment compared to baseline in those treated with DTG administered with TDF/FTC which recovered to baseline levels by week 24, while an increase in folate levels was seen in those treated with DTG administered with tenofovir-alafenamide/FTC [39]. Although these findings do not indicate suppression of maternal folate status by DTG, *in vitro* studies have suggested that DTG is a non-competitive inhibitor of FOLR1 [31], which could impair maternal-fetal folate transport. Partial inhibition of *in vitro* FOLR1-mediated folic acid endocytosis by DTG was also reported in a second study, although this was deemed to be not clinically relevant [30].

Our findings suggest that the low dose of DTG (1x-DTG) did not affect total folate levels or the folate profile in the fetus. However, we observed a small but significant effect on the relative proportions of folates (folate profile) in maternal liver. Our findings of lower methylene tetrahydrofolate as a proportion of total folate appear unlikely to result from impaired activity of dihydrofolate reductase (Dhfr), the enzyme that mediates the reduction of folic acid to DHF and thence to tetrahydrofolate (THF) for use in folate-mediated reactions. DTG does not inhibit Dhfr *in vitro* [31], and we did not detect unmetabolized folic acid in DTG-treated samples. Further studies of the effect of DTG on folate metabolism are needed. For example, our findings of a shift towards lower relative abundance of methylene-THF, encourages investigation of DTG effects on flux through enzymes for which methylene-THF is a product (serine hydroxymethyltransferase 1 or methylenetetrahydrofolate dehydrogenase 1) or substrate (methylenetetrahydrofolate reductase (Mthfr) or thymidylate synthase). Mthfr activity is known to modulate plasma homocysteine [40], higher levels of which have been associated with increased risk of vascular disease [41,42], and could potentially play a role in the fetal vascular defects we observed.

In contrast to 1x-DTG, maternal liver folate profile was not impacted by the higher dose of DTG, but it was notable that total folate levels in the fetus were significantly higher. This suggests that DTG is unlikely to be an inhibitor of fetal folate uptake. The lack of a DTG dose response for fetal anomalies is unexpected, although several compounds have been shown to have such ‘non-monotonic’ dose responses [43]. Most notably non-monotonic relationships have been described for endocrine disruptive compounds and reproductive defects [44]. The fact that non-monotonicity in our study occurred at doses that are not usually tested in regulatory toxicology, suggests that this would not have been identified by mandated pre-clinical testing. Non-monotonicity could result from the induction of compensatory pathways at higher drug doses, that are protective to the fetus [45]. There is evidence that DTG can cross the blood-placental barrier and thus could directly lead to fetal toxicity [46]. Our findings of higher fetal folate levels in the 5x-DTG group compared to both control and 1x-DTG may be indicative of a fetal protective mechanism induced at the higher dose of DTG. Higher fetal folate levels in the 5x-DTG group may be the result of increased folate transport across the placenta, and/or increased uptake of folate into the fetus. It is of interest that a non-monotonic dose response was also observed for the effects of DTG on folate binding to FOLR1 in the presence of human serum albumin and/or calcium [30]. It is also possible that the larger placentas we observed in the 5x-DTG group could have contributed to a larger maternal-fetal exchange surface allowing for greater transport of folate to the fetus. An increase in placental weight could be a result of drug-induced placental hypertrophy, which can serve as a compensatory response in conditions of unfavorable maternal environment or hormonal imbalance [47]. The increase in placental weight in the 5x-DTG group was observed concurrently with a preservation of fetal weight, implying that fetal development was less affected in the 5x-DTG group. This is in contrast to the 1x-DTG group where both placental weight and fetal weight were lower.

Our study has several advantages: its large size, the evaluation of DTG in a clinically relevant dose and ART combination, the initiation of treatment from conception, the inclusion of a concurrent control group, and the inclusion of folate analyses. Our study is limited by the evaluation of primarily external defects, and did not include assessment of commonly observed internal anomalies such as cardiac, renal, or skeletal defects. In addition, mice were treated with DTG in a combination with a dual NRTI backbone which better models the clinical scenario, but makes it more difficult to separate the toxicity effects of DTG from those of the NRTIs. Further, all of our experiments were performed under folate-sufficient conditions which, in addition to the use of the NTD-resistant C57BL/6 mouse strain, may have led to an underestimate of the predisposing effect of DTG for NTDs. Future studies will investigate DTG fetotoxicity under folate-deficient and folate-supplemented conditions.

The potential link between DTG and higher rates of NTDs has created uncertainty among care providers, led to a reduction in use of DTG among women of reproductive age in some countries, and rekindled calls for greater vigilance and more antiretroviral safety studies in pregnancy [[48], [49], [50]]. Our findings, are in agreement with the Botswana results, and provide support for DTG usage in pregnancy being associated with a small increase in NTDs. We also report a non-monotonic relationship between DTG and fetal anomalies that could explain the previous lack of fetal toxicity findings from pre-clinical studies. The association between higher fetal folate levels and fewer fetal anomalies with the higher dose of DTG suggests that higher fetal folate levels may be protective and that folate supplementation may be indicated particularly for women treated with DTG who are considering becoming pregnant.

## Contributors

HM performed all breeding and fetal assessment experiments with help from MGL, EYL, OT and TS. KL performed the mass spectrometry experiments, under the direction of NDEG. LSC performed the magnetic resonance experiments under the direction of JGS. PDO performed the vascular immunohistochemistry experiments. LS and AJC performed the blinded fetal assessments. LS and HM performed all fetal anomaly analyses/interpretation. LS conceived the study and directed the research, with input from AJC and NDEG. HM and LS drafted the manuscript with major contributions from AJC, NDEG and PDO. All authors reviewed the manuscript.

## Declaration of Competing Interest

The authors have no competing interests relating to this study. AJC acts as consultant for ViiV Healthcare Limited, with any fees going to support his research program. LS received personal support for participating in a ViiV organized Think Tank.

## Appendix Supplementary materials

Image, application 1

Supplemental Fig. 1: Representative images of fetal anomalies. Gross morphology of representative fetuses: normal/control **(a)**, mandibular aplasia **(b)**, abdominal wall defect **(c)**, hind limb defect **(d)**, kinked tail **(e)**, curly tail **(f)**, tail flexion **(g)**. Arrows highlight structures with defects. Scale bars = 1 mm. Image, image 2

Supplemental Fig. 2: Fetal folates profile does not differ between treatment arms. Folates profile in whole fetuses collected on gestational day 11.5 and assessed by mass spectrometry. Statistical comparisons by one-way ANOVA with Bonferroni's multiple comparisons post-test. Fetal folate profiles did not differ between groups. *N*=9 fetuses/group from 6 different litters. DHF, dihydropholate; THF, tetrahydrofolate; 5m-THF, 5 methyltetrahydrofolate. Data shown as mean with SEM. Please note that the *y*-axis is shown in 2 segments to permit better visualization of the lower data values. Image, image 3
