# Supplementary figures and images for: Dolutegravir in pregnant mice is associated with increased rates of fetal defects at therapeutic but not at supratherapeutic levels
Source: eBioMedicine. 2020 Dec 18;63:103167. doi: 10.1016/j.ebiom.2020.103167 (PMC7753150; doi:10.1016/j.ebiom.2020.103167)

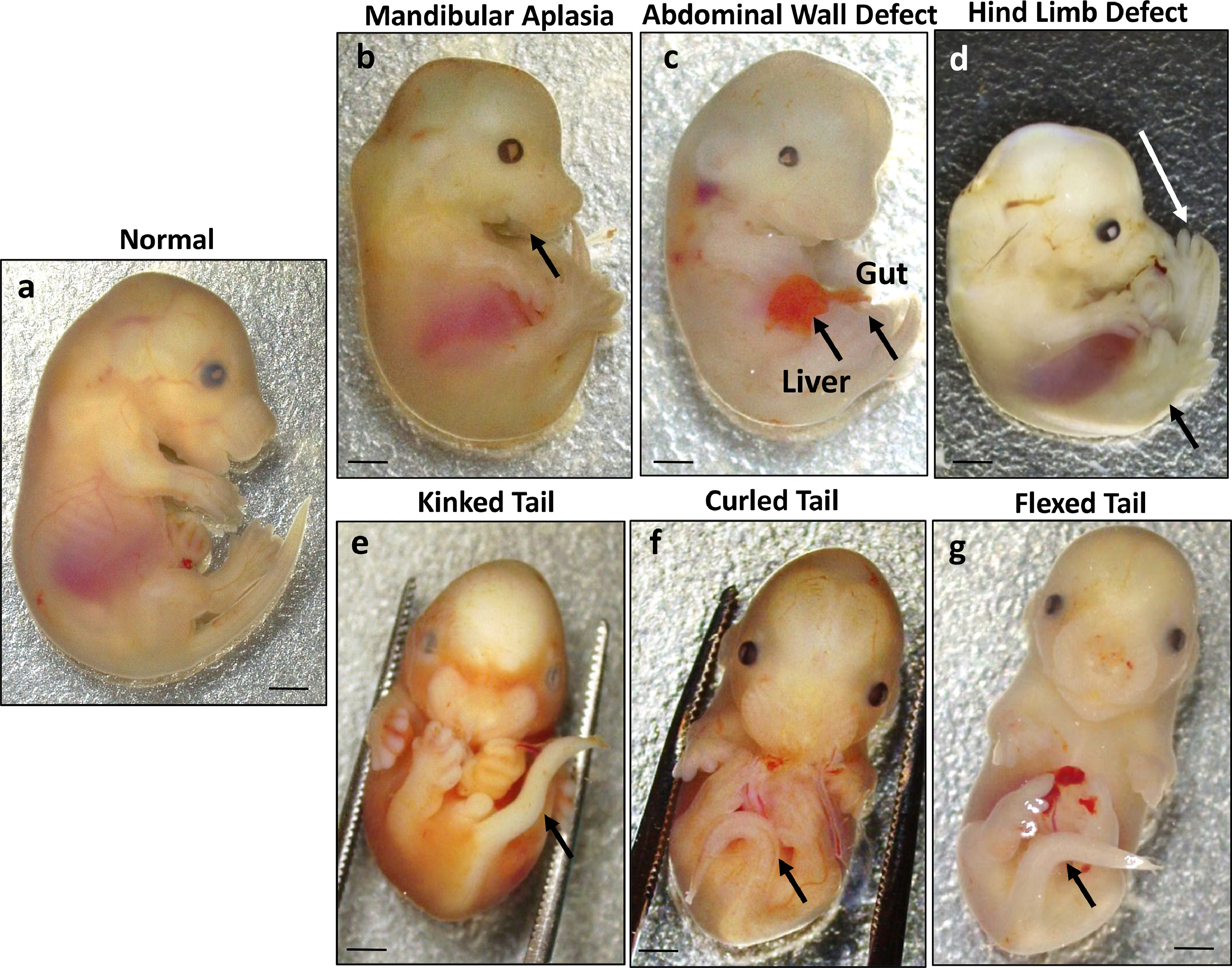

Supplement: Supplementary file 2 — Supplemental Fig. 1: Representative images of fetal anomalies. Gross morphology of representative fetuses: normal/control (a), mandibular aplasia (b), abdominal wall defect (c), hind limb defect (d), kinked tail (e), curly tail (f), tail flexion (g). Arrows highlight structures with defects. Scale bars = 1 mm. [file mmc2.jpg]

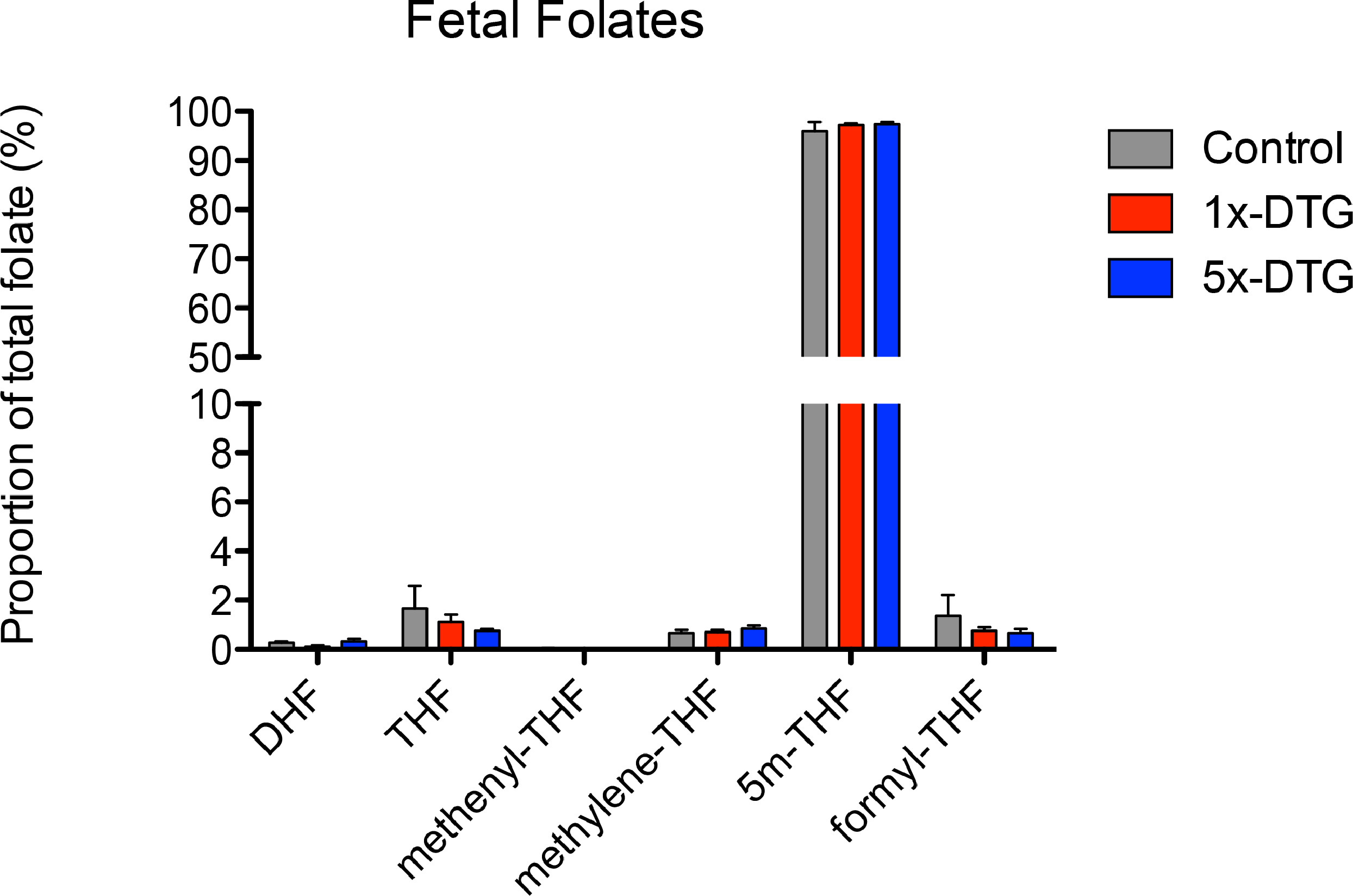

Supplement: Supplementary file 3 — Supplemental Fig. 2: Fetal folates profile does not differ between treatment arms. Folates profile in whole fetuses collected on gestational day 11.5 and assessed by mass spectrometry. Statistical comparisons by one-way ANOVA with Bonferroni's multiple comparisons post-test. Fetal folate profiles did not differ between groups. N=9 fetuses/group from 6 different litters. DHF, dihydropholate; THF, tetrahydrofolate; 5m-THF, 5 methyltetrahydrofolate. Data shown as mean with SEM. Please note that the y-axis is shown in 2 segments to permit better visualization of the lower data values. [file mmc3.jpg]
